# Differences in health care experiences between rare cancer and common cancer patients: results from a national cross-sectional survey

**DOI:** 10.1186/s13023-021-01886-2

**Published:** 2021-06-01

**Authors:** Eline de Heus, Vivian Engelen, Irene Dingemans, Carol Richel, Marga Schrieks, Jan Maarten van der Zwan, Marc G. Besselink, Mark I. van Berge Henegouwen, Carla M. L. van Herpen, Saskia F. A. Duijts

**Affiliations:** 1 Department of Research and Development, Netherlands Comprehensive Cancer Organisation (IKNL), Godebaldkwartier 419, 3511 DT Utrecht, The Netherlands; 2 Department of Medical Oncology, Radboud University Medical Center, Nijmegen, The Netherlands; 3 Dutch Federation of Cancer Patients Organisations (Nederlandse Federatie Van Kankerpatiëntenorganisaties, NFK), Utrecht, The Netherlands; 4 Dutch Breast Cancer Association (Borstkankervereniging Nederland, BVN), Utrecht, The Netherlands; 5 Department of Surgery, Cancer Center Amsterdam, Amsterdam UMC, University of Amsterdam, Amsterdam, The Netherlands; 6 Department of Public and Occupational Health, Amsterdam Public Health Research Institute, Amsterdam UMC, Vrije Universiteit Amsterdam, Amsterdam, The Netherlands

**Keywords:** Expertise, Health care, Hospital choice, Oncology, Patient experience, Rare cancer

## Abstract

**Background:**

Patients with rare cancers face challenges in the diagnostic and treatment phase, and in access to clinical expertise. Since studies on health care experiences of these patients in comparison to patients with more common cancers are scarce, we aimed to explore these differences.

**Methods:**

Data were cross-sectionally collected among (former) adult cancer patients through a national online survey in the Netherlands (October 2019). Descriptive statistics were reported and subgroups (rare vs. common patients) were compared.

**Results:**

In total, 7343 patients (i.e., 1856 rare and 5487 common cancer patients) participated. Rare cancer patients were more often diagnosed and treated in different hospitals compared to common cancer patients (67% vs. 59%, *p* < 0.001). Rare cancer patients received treatment more often in a single hospital (60% vs. 57%, *p* = 0.014), but reported more negative experiences when treated in multiple hospitals than common cancer patients (14% vs. 9%, *p* < 0.001). They also more often received advise from their physician about the hospital to go to for a second opinion (50% vs. 36%, *p* < 0.001), were more likely to choose a hospital specialized in their cancer type (33% vs. 22%, *p* < 0.001), and were more willing to travel as long as necessary to receive specialized care than common cancer patients (55% vs. 47%, *p* < 0.001).

**Conclusions:**

Rare and common cancer patients differ in their health care experiences. Health care for rare cancer patients can be further improved by proper referral to centers of expertise and building a clinical network specifically for rare cancers.

**Supplementary Information:**

The online version contains supplementary material available at 10.1186/s13023-021-01886-2.

## Introduction

Cancer represents the second most common cause of death in Europe [1]. The International Agency for Research on Cancer estimated that there were 3.9 million new European cancer cases in 2018 [2]. In the Netherlands, there were 118,500 new cancer diagnoses in 2019 [3]. About 24% of these are rare cancers, defined as those with an incidence of < 6/100,000 people per year, according to the Surveillance of Rare Cancer in Europe (RARECARE) consortium [4]. In Europe, the five-year survival rates for rare cancers are lower than those for common cancers (49% vs. 63%, respectively) [5]. Therefore, rare cancers pose specific challenges on our health care system, both in the diagnostic and treatment phase, but also regarding access to clinical expertise [6, 7]. In their rare cancer trajectory, patients may be confronted with delayed or wrong diagnoses, conflicting treatment recommendations, logistical difficulties including coordination among multiple physicians and hospitals, and inadequate evidence to guide clinical decision-making [7–10]. Also, some patients with rare cancer (RC) might have longer travel distances in order to receive the necessary and best treatment [11]. Specifically, RC patients might–more than patients with common cancers (CC)–need treatment in centers of expertise (CoE), with multidisciplinary teams focusing specifically on their tumor type.

In the Netherlands, the health care system is based on universal health care access, in which all Dutch residents are entitled to a comprehensive basic health insurance package [12]. The general practitioner (GP) is generally the first access point for patients when they encounter physical complaints. Patients with suspected cancer are referred by the GP to the hospital for diagnosis, staging, and a treatment plan developed in multidisciplinary team meetings. Treatment might take place in the hospital of diagnosis, depending on the type of cancer and patient’s request. However, patients with RC are often referred to a CoE. Moreover, patients might purposely choose for treatment or second opinion in such a hospital as well. After treatment, patients receive follow-up care to check for possible recurrence, to ensure patients’ rehabilitation and to support their quality of life. [13, 14] All Dutch cancer patients are registered in the Netherlands Cancer Registry (NCR) since 1989 [15].

Research regarding experiences of patients with RC within the health care system is limited, and till now, mostly focused on individual types of rare cancers [16, 17]. Since RC patients jointly pose certain known challenges within health care [7–11], we hypothesize that they differ in health care experiences compared to patients with CC. To our knowledge, no explorative study on health care experiences of adult patients with RC has been published so far, and no comparison with experiences of adult patients with CC has been made. Therefore, in a national survey, we aimed to explore differences in health care experiences between patients with RC and patients with CC regarding diagnosis and treatment in multiple hospitals, hospital choice, medical expertise, second opinions, and travel distance to care. Further, objective data from the NCR were used to verify some of the subjective findings.

## Methods

### Study design and participants

A cross-sectional survey was performed among (former) adult cancer patients. Data were collected amongst patients through an explorative national online survey in the Netherlands. The survey was open for two weeks in October 2019. In the survey, participants self-reported their type of cancer by selecting it from a predefined list. The ability of participants to self-report their cancer type accurately was shown to be quite high [18]. The exact classification of a cancer being either rare or common was done afterwards based on the definition of a rare cancer [4] and on the classification used in a previous report on rare cancers in the Netherlands [19].

All participants within this study provided consent, and were informed about privacy policies, in accordance with the General Data Protection Regulation (EU) 2016/679. As they were not involved in an intervention, it was concluded that the Medical Research Involving Human Subjects Act (WMO) does not apply, and according to WMO, ethical approval is not required (2020.257).

### Survey development and content

The explorative online survey was developed by the Dutch Federation of Cancer Patients Organizations (NFK), the Dutch umbrella organization for 19 cancer patient organizations. A project group consisting of a project leader, a researcher, three oncologists (MGB, MIvBH, CMLvH), and five cancer patient organizations’ advocates experienced in quality of care was responsible for the development of the questionnaire’s content, since no validated survey for the aim of this study was available. The final survey (in Dutch) consisted of 29 questions: 27 quantitative and 2 open questions (Additional file 1). In this study, the open questions were not qualitatively analyzed, but used to exemplify experiences of patients. Numerous questions were conditional, i.e., these questions were skipped when irrelevant for the respondent based on previous answers.

The survey started with a selection question to identify respondents who have (had) cancer and three general questions on sociodemographic characteristics. The remaining 25 questions were subdivided into overarching themes: diagnosis and treatment, hospital (choice), second opinion, and traveling to the hospital(s). All questions consisted of multiple answer options, except one question related to the rating of trust in medical expertise, which was scored on a 10-point scale ranging from 1 (no trust at all) to 10 (maximum trust). No personal information of participants was collected, and all data were analyzed anonymously. Only patients who completed the questionnaire at least up to and including the first question on health care experiences were included in the analyses.

### Data collection

Data were collected through the online tool “Survey Monkey” [20]. The questionnaire was nationally distributed through four different channels. First, NFK asked affiliated cancer patient organizations to distribute the survey amongst their members and donors. This was done either directly by mail, or indirectly through their newsletter, website or social media. Second, an invitation was sent to all members of the “Doneer Je Ervaring” (Donate Your Experience) panel comprising (former) cancer patients. Third, an open link to the survey was spread through social media and websites of NFK and some relevant partner organizations (e.g., The Dutch Cancer Society, and website: www.kanker.nl). Last, respondents were actively recruited in several hospitals by means of posters, distribution of flyers, and a movie display in waiting rooms. In the Netherlands, the percentage of inhabitants with Internet access is high, i.e., 97% in 2019 [21]. Objective data was obtained via the NCR and included information on age, gender, type of cancer, number of types of treatment, hospital of diagnosis, hospital of treatment, and the number of hospitals patients were treated in. Hospital of diagnosis and hospital of treatment have been classified according to the Dutch health care system.

### Statistical analyses

Analyses were defined a priori for testing, and applied at patients with RC and CC after data collection. Subgroups were defined based on type of cancer (rare vs. common) [19]. Differences between the subgroups were compared by an independent sample t-test for continuous variables or the Pearson’s chi-square test for categorical variables. Nominal variables are presented as numbers and percentages. Continuous variables are presented as mean and standard deviation (SD) for normally distributed data or median and interquartile range (IQR) for non-normally distributed data. The number of prevalent cases was calculated at the index date of 1^st^ October 2019 (10-year prevalence). For all analyses, a p-value < 0.05 was considered statistically significant. All analyses were performed using IBM SPSS Statistics version 25.

## Results

### Sample characteristics

In total, 8969 participants started the online survey. Of these participants, 556 did not meet the inclusion criteria (i.e., they did not have cancer), 966 did not complete the questionnaire at least up to and including the first question on health care experiences, and 104 could not be classified into a rare or common cancer group or gave duplicate responses. After these exclusions, 7343 participants were eligible for the analysis (i.e., 1856 adult patients with RC and 5487 adult patients with CC) (Table 1).

**Table 1:** Characteristics of the study population by cancer type (total *n* = 7343)

	Rare cancer *n* = 1856	Common cancer *n* = 5487	*P *value
Age in years, mean (SD)	61 (11.9)	63 (10.3)	0.001**
Sex, *n* (%)			0.001**
Male	723 (39%)	1828 (33%)	
Female	1121 (61%)	3645 (67%)	
Educational level, *n* (%)			0.001*
High	781 (43%)	2027 (38%)	
Medium	770 (43%)	2413 (45%)	
Low	261 (14%)	874 (16%)	
Type of cancer, *n* (%)			0.001**
Sarcomas	216 (12%)	0 (0%)	
Female genital organs and breast cancer	284 (15%)	2343 (43%)	
Male genital organ and urological cancer	65 (4%)	1045 (19%)	
Neuroendocrine tumors	28 (2%)	0 (0%)	
Cancer of digestive tract	270 (15%)	1034 (19%)	
Cancer of endocrine organs	84 (5%)	0 (0%)	
Cancer of head and neck	143 (8%)	0 (0%)	
Thoracic cancer	34 (2%)	361 (7%)	
Melanoma of skin and eye	1 (0%)	237 (4%)	
Cancer of central nervous system	50 (3%)	0 (0%)	
Hematological cancer	681 (37%)	467 (9%)	
Current phase of the disease, *n* (%)			0.001**
Cancer-free	904 (54%)	3551 (70%)	
Curable	132 (8%)	489 (10%)	
Incurable	642 (38%)	1056 (21%)	
Number of (types of) treatment^a^, *n* (%)			0.001**
No treatment	58 (3%)	60 (1%)	
1 type of treatment	580 (31%)	1393 (25%)	
2 types of treatment	672 (36%)	1677 (31%)	
> 2 types of treatment	546 (29%)	2357 (43%)	
Years since last treatment, median (range)	2 (0–55 years)	2 (0–56 years)	0.06
Hospital of diagnosis^b^, *n* (%)			0.001**
Academic or cancer-specialized hospital	481 (27%)	714 (13%)	
Top-clinical hospital	811 (45%)	2571 (48%)	
General hospital	520 (29%)	2092 (39%)	
Hospital of treatment^b^, *n* (%)			0.001**
Academic or cancer-specialized hospital	1020 (56%)	1334 (25%)	
Top-clinical hospital	550 (30%)	2431 (45%)	
General hospital	249 (14%)	1597 (30%)	

*n*, number; *SD*, standard deviation

The missing value rate was low (range 0–3%), with one exception, i.e., current phase of disease (8%)

^a^Types of treatment include surgery, radiation, chemotherapy, hormonal therapy, targeted therapy, stem cell transplantation, active surveillance and wait-and-see

^b^Hospital of diagnosis and hospital of treatment have been classified according to the Dutch health care system

^*^p < 0.01

^**^p < 0.001

Patients with RC who participated in the survey were on average younger (61 years, SD 11.9) than patients with CC (63 years, SD 10.3) (*p* < 0.001) (age range 18 to 95 years). Patients with RC were more likely to be men (39% vs. 33%, respectively) (*p* < 0.001) and more often had a high educational level (43% vs. 38%, respectively) (*p* = 0.001) compared to the participating patients with CC. The majority of patients with RC was diagnosed with hematological cancer (37%), female genital organs and breast cancer (15%), or cancer of the digestive tract (15%), while CC patients were mostly diagnosed with female genital organs and breast cancer (43%), male genital organ and urological cancer (19%), or cancer of the digestive tract (19%) (*p* < 0.001). Most patients with RC received two types of treatment (36%), while most patients with CC received more than two types of treatment (43%) (*p* < 0.001). Finally, patients with RC were more often in an incurable stage of the disease at time of survey completion compared to patients with CC (38% vs. 21%, respectively) (*p* < 0.001) (Table 1). A selection of responses on the qualitative questions can be found in Table 2.

**Table 2:** Selection of illustrative quotes from rare cancer patients

Topic	Quotes
Experiences regarding diagnosis and treatment in multiple hospitals	“Bad communication from hospital X to hospital Y. Information was regularly missing, which almost led to crucial mistakes regarding treatment several times.”
Experiences regarding hospital choice	“It is difficult to find out if another hospital would be better. You get into a crazy merry-go-around in the hospital where the diagnosis is made. Then you only want one thing, and that is to start treatment as soon as possible.”
Experiences regarding medical expertise and second opinions	“The pathologist of hospital X asked for a second opinion himself, because of the rarity of angiosarcomas and thus I was immediately referred to hospital Y.”
Experiences regarding travel distance to care	“If your life is at stake and you want maximum care, travel time is a secondary problem to be solved.”

### Experiences regarding diagnosis and treatment in multiple hospitals

Patients with RC more often received their diagnosis and treatment in different hospitals compared to patients with CC (67% [95% CI 65–69] vs. 59% [95% CI 57–60], respectively) (*p* < 0.001). Diagnosis most often took place in a top-clinical hospital for both RC and CC patients (45% [95% CI 43–47] vs. 48% [95% CI 46–49], respectively) (*p* < 0.001), while treatment for patients with RC mostly took place in an academic or cancer-specialized hospital (56% [95% CI 54–58]) and in a top-clinical hospital for patients with CC (45% [95% CI 44–47]) (*p* < 0.001). For patients with CC, the hospital of diagnosis more often continued to remain their first point of contact and their treating hospital during the whole cancer trajectory compared to patients with RC (78% [95% CI 77–79] vs. 61% [95% CI 58–63], *p* < 0.001).

Focusing specifically on treatment, a significant difference was found between RC and CC patients regarding the number of hospitals they were treated in (*p* = 0.014). Patients with RC were more often treated in one hospital compared to patients with CC (60% [95% CI 58–62] vs. 57% [95% CI 55–58], *p* = 0.014). In case patients with RC were treated in multiple hospitals, they reported more negative experiences than patients with CC (14% [95% CI 12–17] vs. 9% [95% CI 8–10], *p* < 0.001). That is, patients with RC more often than patients with CC indicated that they did not feel supported by their physician when referred to another hospital (19% [95% CI 16–22] vs. 16% [95% CI 15–18], *p* = 0.024), that their medical files were not available on time in the other hospital (18% [95% CI 15–21] vs. 13% [95% CI 12–15], *p* = 0.001), and that their health care providers were not well informed about their situation (18% [95% CI 15–21] vs. 15% [95% CI 13–17], *p* = 0.046).

### Experiences regarding hospital choice

More than half of the RC and CC patients (51% [95% CI 48–53] vs. 52% [95% CI 51–53], respectively) never thought about the most suitable hospital regarding their cancer treatment (*p* = 0.424). Of the patients who did think about which hospital was most suitable for them, 73% ([95% CI 70–76]) of the patients with RC and 71% ([95% CI 69–73]) of the patients with CC indicated that they have searched for information and/or have discussed this with someone (*p* = 0.313). Patients with RC were more likely to choose a hospital, because it was specialized in their type of cancer than patients with CC (33% [95% CI 31–35] vs. 22% [95% CI 21–24], *p* < 0.001). In retrospect, one in every six patients with RC (16% [95% CI 14–18]) and one in every five patients with CC (20% [95% CI 19–21]) would have done something in a different way regarding their choice of treatment hospital for their type of cancer (*p* < 0.001), such as figuring out better what the best hospital for their type of cancer was or asking for a second opinion.

### Experiences regarding medical expertise and second opinions

Differences between RC and CC patients were found regarding trust in medical expertise concerning their treatment. That is, respectively 66% ([95% CI 64–68]) and 61% ([95% CI 60–63]) gave, on a 0–10 scale, an ‘excellent’ score (range 9–10), 30% ([95% CI 29–31]) and 35% ([95% CI 34–35]) gave a ‘sufficient to good’ score (range 6–8), and 4% of both RC and CC patients gave an ‘insufficient’ score (range 1–5) ([95% CI 3–4]; [95% CI 4–4], respectively) (*p* = 0.004). Further, patients with RC had slightly more often a second opinion compared to patients with CC (23% [95% CI 21–25] vs. 22% [95% CI 21–23], *p* = 0.211). Patients with RC more often indicated to have been advised by their physician about the hospital to go to for a second opinion, compared to patients with CC (50% [95% CI 45–55] vs. 36% [95% CI 33–39], *p* < 0.001).

### Experiences regarding travel distance to care

Patients with RC were more often willing to travel as long as necessary to receive care from a hospital specialized in their cancer type in comparison to patients with CC (55% [95% CI 53–58] vs. 47% [95% CI 45–48], *p* < 0.001). Patients with CC were more likely to choose a hospital close to home than patients with RC (65% [95% CI 64–67] vs. 46% [95% CI 44–49], *p* < 0.001). Patients with RC (54% [95% CI 51–56]) more often travelled half an hour or longer to the hospital of treatment than patients with CC (35% [95% CI 33–36]) (*p* < 0.001). Furthermore, significant differences were found between RC and CC patients regarding their travel experience; 67% ([95% CI 65–69]**)** of the patients with RC indicated that they never experienced problems with travelling compared to 79% ([95% CI 78–80]) of the patients CC (*p* < 0.001). Of the patients who had problems with travelling to the hospital, both RC and CC patients explained that they were (sometimes) too sick or in too much pain (14% [95% CI 12–15] vs. 8% [95% CI 8–9], respectively), considered it as a burden to travel to the hospital for treatment frequently (14% [95% CI 12–15] vs. 8% [95% CI 7–9], respectively), and experienced it as a burden for the ones who came with them (12% [95% CI 10–13] vs. 7% [95% CI 6–7], respectively) (all *p* < 0.001).

### Comparison of cancer registry and survey data

With respect to gender, data from the NCR showed that RC and CC patients are more often male (48% and 49%, respectively) compared to RC and CC patients who participated in the survey (39% and 33%, respectively) (Table 3). Patients with RC in the NCR are less often diagnosed with hematological cancer than patients in the survey (18% vs. 37%, respectively), while patients with CC in the NCR are less often diagnosed with female genital organs and breast cancer than patients in the survey (26% vs. 43%, respectively). Furthermore, both RC and CC patients in the NCR (45% and 40%, respectively) receive more often one type of treatment than participating patients in the survey (31% and 25%, respectively). According to the NCR data, the hospital of treatment for patients with RC is less often an academic or cancer-specialized hospital when compared to the survey (43% vs. 56%, respectively). In addition, diagnosis and treatment of patients with RC and patients with CC more often take place in one hospital according to the NCR data (52% and 64%, respectively), compared to data resulting from the survey (33% and 42%, respectively).

**Table 3:** Comparison of survey and NCR data (10-year prevalence) for rare and common cancer patients

	Rare cancer		Common cancer	
	Survey	NCR	Survey	NCR
Age in years, mean	61	58	63	65
Gender, %				
Male	39%	47%	33%	48%
Female	61%	53%	67%	52%
Type of cancer, %				
Sarcomas	12%	8%	0%	0%
Female genital organs and breast cancer	15%	20%	43%	26%
Male genital organ and urological cancer	4%	10%	19%	22%
Neuroendocrine tumors	2%	7%	0%	0%
Cancer of digestive tract	15%	6%	19%	17%
Cancer of endocrine organs	5%	5%	0%	0%
Cancer of head and neck	8%	16%	0%	0%
Thoracic cancer	2%	2%	7%	6%
Melanoma of skin and eye	0%	3%	4%	24%
Cancer of central nervous system	3%	5%	0%	0%
Hematological cancer	37%	18%	9%	7%
Number of (types of) treatment^a^, %				
No treatment	3%	4%	1%	6%
1 type of treatment	31%	46%	25%	40%
2 types of treatment	36%	28%	31%	30%
> 2 types of treatment	29%	22%	43%	24%
Hospital of diagnosis, %				
Academic or cancer-specialized hospital	27%	17%	13%	8%
Top-clinical hospital	45%	48%	48%	51%
General hospital	29%	36%	39%	41%
Hospital of treatment, %				
Academic or cancer-specialized hospital	56%	43%	25%	12%
Top-clinical hospital	30%	37%	45%	51%
General hospital	14%	20%	30%	37%
Diagnosis and treatment in one hospital, %				
Yes	33%	51%	42%	64%
No	67%	49%	59%	36%
Number of hospitals patients were treated in, %				
1	60%	80%	57%	74%
≥ 2	40%	20%	43%	26%

^a^ Types of treatment include surgery, radiation, chemotherapy, hormonal therapy, targeted therapy, stem cell transplantation, active surveillance and wait-and-see

## Discussion

### Main findings

The aim of this study was to explore possible differences in health care experiences between patients with RC and patients with CC. Our results indeed showed differences between these two adult patient groups. Patients with RC are more often diagnosed and treated in different hospitals compared to patients with CC. Treatment more often takes place in one hospital for patients with RC, but if treatment takes place in multiple hospitals, they experience this as more negative than patients with CC. Patients with RC are more often advised by their physician about the hospital to go to for a second opinion than patients with CC. In addition, RC patients are more likely to choose a hospital specialized in their cancer type, while CC patients are more likely to choose a hospital close to home. Finally, patients with RC are more often willing to travel as long as necessary to receive care from a specialized hospital in comparison to patients with CC.

### Interpretation of findings

Our study showed that diagnosis and treatment of patients with RC mostly take place in different hospitals. This finding is in line with previous literature on rare cancers. Scandinavian registry studies revealed, for example, that nearly all patients, after being diagnosed with bone sarcoma (derived from the Scandinavian Sarcoma Register [22]) or soft-tissue sarcoma (derived from the Swedish Cancer Registry [23]) are referred to a sarcoma expert center for their treatment [22, 23]. This implies that most patients with RC in our survey are referred to another hospital for treatment in case the hospital of diagnosis is lacking expertise for the treatment of the specific rare cancer type. While such a treatment decision may benefit the patient, it also may lead to fragmentation of care [24].

Focusing specifically on treatment, patients with RC more frequently receive this care in a single hospital compared to patients with CC, probably indicating a certain level of centralization of care for those with a rare tumor type. This is in line with the study by Gatta et al. (2017) on patients with RC, diagnosed in 2000–2007, in seven European countries [5]. Although centralization of care was not completely realized at the time of the study, the authors indicated that the highest centralization patterns were found in Slovenia and in the Netherlands. For example, care for patients diagnosed with bone sarcoma was already highly centralized between 2000 and 2007 in the Netherlands, i.e., 75% of these patients were seen in only five hospitals. Nowadays, care for these patients is even centralized in four bone tumor centers [25]. Still, while in almost every hospital treatment for CC patients is offered, only a few designated CoE exist in which optimal treatment for patients with RC is available [5, 26]. This centralization of care has been shown to improve disease outcomes for rare cancers [27, 28].

With regard to being treated in multiple hospitals, patients with RC had more negative experiences than patients with CC. Although speculative, negative experiences of being treated in multiple hospitals might, among others, be explained by delays in care caused by patient referral from one to another hospital [29, 30]. Studies showed that these delays may result in major psychosocial worries and dissatisfaction with the health care system [31–33]. Moreover, negative experiences may be more prevalent in patients with RC compared to patients with CC, since the former often lack a clear cancer care pathway due to fragmentation of care [16, 34, 35].

Regarding second opinion, patients with RC were more often recommended by their physician about the hospital to go to for such a second opinion than patients with CC. A possible explanation for this may be related to the confidence of physicians with offering specific care for patients with RC. For a limited number of rare cancers, centralization of care is present, because of which these physicians are aware of the hospital that provides the best care for this patient. Previous studies on second opinions in breast cancer patients showed that physicians specifically inform those patients who are highly educated and more involved in the decision-making process, and these patients were also more likely to request a second opinion [36, 37]. Accordingly, patients with RC in our study had a higher level of education than CC patients, and thus might be more inclined to learn about second opinion options, or request such an opinion themselves.

Considering hospital choice, patients with RC were more likely than patients with CC to choose a hospital with expertise regarding their cancer type. However, for patients with RC, it remains often unclear where the expertise for their specific cancer type is available due to fragmentation of care. Moreover, CC patients were more likely to choose a hospital close to home than RC patients, but expertise for those patients is in general more accessible close to home. Consequently, patients with RC experience longer travel distances to receive specialized care, but they also showed greater willingness to travel for this specialized care. Previous studies in head and neck cancer patients found, in line with our findings, that those patients were willing to travel significant distances to ensure access to better cancer care [38, 39]. Regardless of the travel distance to the hospital, patients with RC seem to deliberately search for the best available cancer care, while patients with CC have a lower incentive to search for better care beyond their regional hospital.

### Limitations and strengths

A strength of the present study is that, to the best of our knowledge, this is the first explorative study showing differences in health care experiences between patients with RC and CC. Other strengths of this study are the comparison of the survey data with cancer registry data from all Dutch cancer patients, which enabled the researchers to investigate the generalizability of the study results, and the large sample size. Yet, results should be interpreted with caution, as statistically significant differences in such a large sample size might not always be clinically relevant.

Several limitations of this study need to be addressed as well. First, participants were mainly recruited through cancer patient organizations and might therefore not be representative for all cancer patients. That is, patients with hematological cancers were overrepresented in the survey. Also, patients related to these organizations often have a higher educational level, which was found in our study as well. Second, although we included a broad sample of participants, the number of RC and CC patients who chose not to complete the survey is unknown, which might have resulted in participation bias. Third, in this study, no data was collected on year of diagnosis, relapse status, and whether patients changed hospitals at their own request or through active referral. Not having gathered data on these items may have influenced our interpretation of the experiences patients reported in this study. Fourth, the questionnaire was only available in Dutch and no psychometric properties were tested. Fifth, cancer diagnosis was self-reported, and the classification of a cancer being either rare or common was done in retrospect, which might have led to misclassification of patients. Sixth, it should be emphasized that our cross-sectional study merely established associations, and no causal relations. Finally, regarding generalizability, one should be aware that the Dutch health care system and degree of centralization might differ from other countries. On a global level, centralization of rare cancer care is still suboptimal, with the exception of a country such as France where clear organization of rare cancer care exists [40]. Due to these limitations, findings should be interpreted with caution.

### Implications for research and clinical practice

Future research on rare and common cancers should classify the cancer type beforehand, using the list of rare cancers as comprised by RARECARENet [41]. Also, researchers should aim to include a generic group of patients with RC and CC in future studies, i.e., a higher percentage of male and low educated patients in our study sample would have given a more accurate representation of the overall group of cancer patients. Further, longitudinal studies on health care experiences between RC and CC patients should be conducted to enable assessment of causal relationships. Finally, in order to reduce heterogeneity, researchers should further examine differences between patients with RC and CC by site.

With regard to clinical practice, health care providers should be aware of the different health care experiences of patients with RC and CC. They should take into account the experiences of patients with RC when referring them to another hospital for treatment (e.g., if possible, they should refer them to a CoE and/or support a patient’s request for a second opinion). Also, they should offer them appropriate guidance and support to reduce their negative experiences when treated in multiple hospitals. In addition, observed differences in health care experiences between patients with RC and CC could be reduced by establishing regional clinical networks and ensuring appropriate care to all rare cancer patients regardless their point of access. Such a network could, among others, simplify and accelerate referrals, which may diminish challenges patients with RC are facing during their patient journey. Health care providers can play an important role in this by developing a clear patient pathway, giving support during the whole cancer trajectory and, if necessary, proper referral to CoE. Herewith, they support similar access and continuity of health care for both patients with RC and CC.

## Conclusion

The results of this study showed that differences in health care experiences between adult patients with RC and CC exist. Regional clinical networks should be established to support proper referral of patients with RC to centers of expertise, and to improve their care. Future longitudinal studies are needed to determine the causal relationship between care and health-related outcomes in patients with RC.

## Supplementary Information

**Additional file 1: Appendix I**. Survey.

## Data Availability

The data that support the findings of this study are available from NFK. Restrictions apply to the availability of these data, which were used under license for this study. Data are available with the permission of NFK.
