# Supplementary material for: Differences in health care experiences between rare cancer and common cancer patients: results from a national cross-sectional survey
Source: Orphanet J Rare Dis. 2021 Jun 1;16:249. doi: 10.1186/s13023-021-01886-2 (PMC8170927; doi:10.1186/s13023-021-01886-2)
Supplement: Supplementary file 1 — Additional file 1: Appendix I. Survey. [file 13023_2021_1886_MOESM1_ESM.docx]

**Differences in health care experiences between rare cancer and common cancer patients: results from a national cross-sectional survey**

Orphanet Journal of Rare Diseases

**Appendix I. Survey.**

1. This questionnaire is intended for people who have (had) cancer. Does this apply to you?
2. Yes, I have (had) cancer
3. No

**About you**

1. What is your sex?
2. Male
3. Female
4. Other
5. What is your year of birth? …
6. What is your highest level of education?
7. No education achieved
8. Primary school (primary education)
9. Lower vocational secondary education (e.g. LTS, LHNO, huishoudschool, VMBO-basis beroepsgericht, VMBO-kader beroepsgericht, LEAO)
10. Secondary general education (e.g. ULO, MULO/MAVO, 3 jaars-HBS, VMBO-T)
11. Secondary vocational education (MBO)
12. Senior general secondary education/pre-university education (e.g. HAVO, VWO, gymnasium, HBS, MMS)
13. Higher professional education (HBO, bachelor, post-HBO)
14. University education (university, master, doctoral degree)
15. I would rather not say
16. Otherwise, namely

**About your disease and treatment**

1. Which type of cancer do/did you have?

*If you have (had) more types of cancer, then fill in the most recent type of cancer. Pay attention: the type of cancer you fill in here, will appear during the rest of the questionnaire.*

1. Which treatment(s) did you have for [Q5]?

*Multiple answers possible.*

1. Surgery
2. Chemotherapy
3. Radiation
4. Hormonal therapy
5. Immunotherapy
6. Targeted therapy
7. Stem cell transplantation
8. Wait and see
9. Active surveillance or watchful waiting
10. Stoma placement
11. Pain-relieving treatment
12. I do not know which treatment I have had
13. I did not have any treatment
14. Otherwise, namely …
15. In which year did your most recent treatment* for [Q5] take place?

** By treatment we mean: surgery, chemotherapy, radiation, hormonal therapy, immunotherapy, targeted therapy, stem cell transplantation, wait and see, active surveillance or watchful waiting, stoma placement or pain-relieving treatment.*

1. Which of the following descriptions matches your situation (at this moment) the most, regarding [Q5]?
2. I (probably) do not have cancer anymore
3. I will (probably) get better
4. I will (probably) not get better
5. I do not know/not applicable

**About your hospital(s)**

1. In which hospital was your diagnosis [Q5] made? …
2. In which hospital were you treated* for [Q5]?

*Have you been treated in several hospitals? Then assume the hospital that was your first point of contact** for your treatment.*

** By treatment we mean: surgery, chemotherapy, radiation, hormonal therapy, immunotherapy, targeted therapy, stem cell transplantation, wait and see, active surveillance or watchful waiting, stoma placement or pain-relieving treatment.*

*** By the hospital that is your first point of contact for your treatments, we mean the hospital where you have the most checks and conversations. The medical specialist, who has an overview of all your treatments as well as treatments in other hospitals, also works in this hospital.*

1. In how many hospitals* in total have you been treated** for [Q5]?
2. One hospital
3. Two hospitals
4. Three or more hospitals

** One hospital can have multiple locations, e.g., VieCuri Medisch Centrum Venlo and VieCuri Medisch Centrum Venray. We regard this as one hospital. Therefore, this question concerns different hospitals and not different locations of the same hospital.*

** *By treatment we mean: surgery, chemotherapy, radiation, hormonal therapy, immunotherapy, targeted therapy, stem cell transplantation, wait and see, active surveillance or watchful waiting, stoma placement or pain-relieving treatment.*

1. How did you experience treatment in multiple hospitals for [Q5]?
2. I (mostly) experienced this as positive
3. I have not experienced this as positive nor as negative
4. I (mostly) experienced this as negative
5. I do not know/not applicable
6. What have you experienced (mostly) as positive or (mostly) as negative about the fact that you were treated in two or more hospitals for [Q5]?

…

1. You have been treated in multiple hospitals for [Q5]. To what extent have you experienced the arguments below (in general)?

*Answer possibilities: Always, mostly, sometimes, never, I do not know/not applicable*

- I felt supported by my doctor(s) in one hospital, when I was referred to another hospital for (a part of my) treatment
- My file or medical research results in one hospital were on time available in the other hospital
- My health care providers in one hospital were well informed of what happened to me in the other hospital
- I knew in which hospital I had to be with questions or problems

Give an explanation if necessary: …

**Second opinion**

1. Have you had a second opinion* for [Q5]?

*Multiple answers possible.*

1. Yes, shortly after my diagnosis, but before I started my first treatment
2. Yes, later in my illness, during or after my treatment(s)
3. No
4. I do not know/not applicable

** With a second opinion, another doctor (in another hospital) will look at your diagnosis and treatment options again. You can request a second opinion if you want more certainty about your diagnosis or treatment options.*

1. You have had a second opinion for [Q5] once or multiple times. To what extent have you experienced the arguments below (in general)?

*Answer possibilities: Always, mostly, sometimes, never, I do not know/not applicable*

- I felt supported by my doctor(s) in one hospital, when I went to another hospital for a second opinion
- The doctor in one hospital advised me a hospital to go to for a second opinion
- My file or medical research results in one hospital were on time available in the hospital of the second opinion

Give an explanation if necessary: …

**About the choice for your hospital**

1. Have you ever thought about which hospital is most suitable for you for the treatment of [Q5]?
2. Yes
3. No
4. I do not know/not applicable

Give an explanation if necessary: …

1. Have you searched for information and/or discussed with someone to find out which hospital for you is most suitable for treatment of [Q5]?
2. Yes
3. No
4. I do not know/not applicable
5. Where did you search for information and/or with whom did you discuss to find out which hospital for you is most suitable for treatment of [Q5]?

*Multiple answers possible.*

1. Website hospital
2. Website cancer patient organization
3. Website health care insurer
4. Other website(s)
5. A decision aid* completed on the internet
6. Flyer hospital
7. Flyer cancer patient organization
8. Discussed with my general practitioner
9. Discussed with hospital
10. Discussed with cancer patient organization
11. Discussed with health care insurer
12. Discussed with fellows/acquaintances
13. I do not know/not applicable
14. Otherwise, namely …

** A decision aid is an instrument on the internet that helps you choose a hospital that suits you the most. The decision aid asks you several questions and you indicate what is important to you. Thereafter, you receive an overview of hospitals that suit your wishes the most. You can then compare the hospitals.*

1. What were your reasons for choosing hospital [Q10] for the treatment of [Q5]?

*Multiple answers possible.*

1. This hospital was close to home
2. The travel and/or parking costs for this hospital were low
3. I was already being treated for another disease in this hospital
4. I already knew this hospital
5. I had a doctor at this hospital with whom I felt comfortable
6. This hospital seemed good to me
7. By information I found on the internet, this hospital seemed most suitable for me
8. My general practitioner recommended this hospital to me
9. My doctor in another hospital recommended this hospital to me
10. The cancer patient organization recommended this hospital to me
11. My health care insurer recommended this hospital to me
12. Fellows/acquaintances recommended this hospital to me
13. This hospital is specialized in [Q5]
14. I have had a second opinion in this hospital
15. In this hospital I could get a specific treatment, which I could not get in the other hospital (e.g. surgery or participating in a trial)
16. My other hospital could not (further) help me and this hospital could
17. There was no specific reason why I chose this hospital
18. I do not know/not applicable
19. Otherwise, namely …
20. How much trust do you have in the medical expertise of hospital [Q10] when it comes to treatment of [Q5]?

Give a score between 1 and 10 (1=no trust at all – 10=maximum trust, I do not know/not applicable): …

Give an explanation if necessary: …

1. Do you know which hospital(s) is/are specialized in [Q5]?
2. Yes
3. No

Give an explanation if necessary: …

**About travelling to your hospital**

1. What was your travel time* (one-way) to [Q10], when you were treated for [Q5]?
2. Less than half an hour
3. Between half an hour and 1 hour
4. Between 1 hour and 1,5 hours
5. Between 1,5 and 2 hours
6. Between 2 and 3 hours
7. More than 3 hours

** By travel time we mean: the time you travel from home to hospital (one-way). Without time that you spend in the parking garage or for walking to the outpatient clinic.*

1. How did you experience travelling* to [Q10], when you were treated for [Q5]?
2. I did not have a problem with travelling
3. I sometimes had a problem with travelling
4. I often had a problem with travelling
5. I always had a problem with travelling

** By travelling we mean: the travel distance, the travel time and the comfort of travelling.*

1. Why was travelling to [Q10], when you were treated for [Q5] (to a greater or lesser extent) a problem for you?

*Multiple answers possible.*

1. I was (sometimes) too ill or I (sometimes) had too much pain to travel
2. I often had to go to this hospital for treatment
3. The travel distance and/or travel time to the hospital was too long
4. The travel and/or parking costs were too high for me
5. I had to go to the hospital on my own, nobody could come with me
6. I do not have my own transportation
7. I thought it was a burden for my loved ones who came with me
8. The hospital was too far away for my loved ones, so I received little or no visit in the hospital
9. I do not know/not applicable
10. Otherwise, namely …
11. How long would you be willing to travel (one-way) for care from a hospital that is specialized in [Q5]?

*Indicate your maximum travel time*.*

1. Half an hour maximum
2. 1 hour maximum
3. 1,5 hours maximum
4. 2 hours maximum
5. 3 hours maximum
6. There is no maximum travel time. I will travel as long as necessary to receive the care of a hospital that is specialized in [Q5]
7. I do not know/not applicable

Give an explanation if necessary: …

** By travel time we mean: the time you travel from home to hospital (one-way). Without time that you spend in the parking garage or for walking to the outpatient clinic.*

**If you could redo it…**

1. In retrospect, would you have done something else if it concerns the choice for [Q10] for the treatment of [Q5]?

*Multiple answers possible.*

1. Yes, I would have found out (better), which is the right hospital for me
2. Yes, I would have discussed (more) with my general practitioner, which is the right hospital for me
3. Yes, I would have discussed (more) with my doctor in the hospital of diagnosis, which is the right hospital for me
4. Yes, I would have discussed (more) with a cancer patient organization, which is the right hospital for me
5. Yes, I would have discussed (more) with my health care insurer, which is the right hospital for me
6. Yes, I would have discussed (more) with fellows/acquaintances, which is the right hospital for me
7. Yes, I would have done a second opinion (earlier)
8. Yes, I would not have gone to this hospital
9. Yes, I would have chosen a different hospital (earlier), namely a hospital that is specialized in [Q5]
10. Yes, I would have chosen a different hospital (earlier), namely a hospital closer to home
11. Yes, I would have chosen a different hospital (earlier), namely a hospital where I was already being treated for another disease
12. Yes, I would have chosen a different hospital (earlier), namely a hospital that I already know
13. No, I would not have done something else
14. I do not know/not applicable
15. Otherwise, namely …

**Finally**

1. Finally, is there anything you would like to share?

*To protect your privacy, we ask you to not fill in any personal information.*

…

1. We would like to improve our Donate Your Experience. It helps us to know how people get into our questionnaires. How did you get into this questionnaire?

*Multiple answers possible.*

1. I am member of the Donate Your Experience panel
2. Through a cancer patient organization
3. Through a hospital
4. Through the general practitioner
5. Through social media
6. Through Gezondheidsplein.nl or dokterdokter.nl
7. Through Kanker.nl
8. Through KWF (social media/website)
9. Through an online advertisement
10. Through family/friends/acquaintances
11. Other
